# In vitro assessment of probiotic attributes for strains contained in commercial formulations

**DOI:** 10.1038/s41598-022-25688-z

**Published:** 2022-12-14

**Authors:** Diletta Mazzantini, Marco Calvigioni, Francesco Celandroni, Antonella Lupetti, Emilia Ghelardi

**Affiliations:** 1 Department of Translational Research and New Technologies in Medicine and Surgery, University of Pisa, Pisa, Italy; 2 Research Center Nutraceuticals and Food for Health-Nutrafood, University of Pisa, Pisa, Italy

**Keywords:** Microbiology, Medical research

## Abstract

Although probiotics are often indiscriminately prescribed, they are not equal and their effects on the host may profoundly differ. In vitro determination of the attributes of probiotics should be a primary concern and be performed even before clinical studies are designed. In fact, knowledge on the biological properties a microbe possesses is crucial for selecting the most suitable bacteriotherapy for each individual. Herein, nine strains (*Bacillus clausii* NR, OC, SIN, T*, Bacillus coagulans* ATCC 7050, *Bifidobacterium breve* DSM 16604, *Limosilactobacillus reuteri* DSM 17938, *Lacticaseibacillus rhamnosus* ATCC 53103, and *Saccharomyces boulardii* CNCM I-745) declared to be contained in six commercial formulations were tested for their ability to tolerate simulated intestinal conditions, adhere to mucins, and produce β-galactosidase, antioxidant enzymes, riboflavin, and d-lactate. With the exception of *B. breve*, all microbes survived in simulated intestinal fluid. *L. rhamnosus* was unable to adhere to mucins and differences in mucin adhesion were evidenced for *L. reuteri* and *S. boulardii* depending on oxygen levels. All microorganisms produced antioxidant enzymes, but only *B. clausii*, *B. coagulans*, *B. breve*, and *L. reuteri* synthesize β-galactosidase. Riboflavin secretion was observed for *Bacillus* species and *L. rhamnosus*, while d-lactate production was restricted to *L. reuteri* and *L. rhamnosus*. Our findings indicate that the analyzed strains possess different in vitro biological properties, thus highlighting the usefulness of in vitro tests as prelude for clinical research.

## Introduction

Probiotics are defined as “live microorganisms that, when administered in adequate amounts, confer a health benefit on the host”^1^. Several lactic acid bacteria belonging to the genera *Lactobacillus, Lacticaseibacillus*, *Lactiplantibacillus*, *Lentilactobacillus*, *Levilactobacillus*, *Ligilactobacillus*, and *Limosilactobacillus* (a.k.a. *Lactobacillus*), *Streptococcus*, and *Enterococcus*, as well as some *Bifidobacterium* and spore-forming *Bacillus* species, have been shown to possess a plethora of in vitro and in vivo beneficial attributes, being progressively included in a great number of probiotic formulations commercialized worldwide^2,3^. In addition, the yeast *Saccharomyces cerevisiae* has increasingly taken on as probiotic microorganism in the last decades^4^.

Orally administered probiotics are commonly used for preventing and/or treating some gastrointestinal conditions linked to an unbalanced gut flora or gut barrier alteration, including antibiotic-associated, traveler, and acute-infectious diarrhea, *Clostridium difficile* infection, inflammatory bowel diseases, and irritable bowel syndrome^3,5^. In addition, the use of probiotics for the treatment of some other conditions (e.g. allergy, osteoporosis, lactose intolerance, metabolic syndromes, as well as neurological, cardiovascular, respiratory, and liver diseases) has progressively been documented^3^.

Probiotics can transit through and eventually colonize the gastrointestinal environment to exert their beneficial effects on host health^6,7^. Several reports highlighted the ability of probiotics to enhance gut barrier function and host immune response, and to reduce the oxidative stress due to accumulation of oxygen reactive species (ROS)^5,8,9^. Probiotic microorganisms can compete with pathogens for nutrients and mucosal binding sites, as well as produce antimicrobial molecules, thus counteracting infections by pathogenic organisms^5^. Probiotics can also produce vitamins and food-degrading enzymes that can be helpful during digestion and in compensating vitamin deficiencies^3,10^. The production of riboflavin by probiotics is an attractive issue since riboflavin deficiency (i.e. ariboflavinosis), derived from diets lacking riboflavin-rich products, currently represents the most common vitamin deficiency in developing countries^11^. Besides their beneficial properties, probiotic strains can also synthesize molecules (e.g. d-lactate) potentially exerting negative effects in some individuals^12–14^.

Since probiotic properties are often species- or strain- specific^15^, knowledge on the peculiar properties of strains is crucial for choosing probiotic therapies based on specific individual needs.

The present study aimed at evaluating and comparing some in vitro properties of nine microbial strains isolated from commercial formulations sold as containing probiotics. In particular, the ability of these microbes to survive in simulated intestinal fluid, adhere to mucins, produce antioxidant enzymes (i.e. catalase and superoxide dismutase), riboflavin (i.e. vitamin B2), lactase (i.e. β-galactosidase), and d-lactate were investigated. The in vitro analysis of probiotic attributes of microbes isolated from preparations present on the market can be helpful in elucidating the potential beneficial effects provided by these formulations in vivo.

## Results

### Survival of probiotic microorganisms in simulated intestinal fluid

*Bacillus clausii* strains NR, OC, SIN, and T, *Bacillus coagulans* ATCC 7050, *Bifidobacterium breve* DSM 16604, *Limosilactobacillus reuteri* DSM 17938*, Lacticaseibacillus rhamnosus* ATCC 53103, and *Saccharomyces boulardii* CNCM I-745, declared to be contained in six commercial formulations were isolated and used throughout this study.

**Figure 1:**
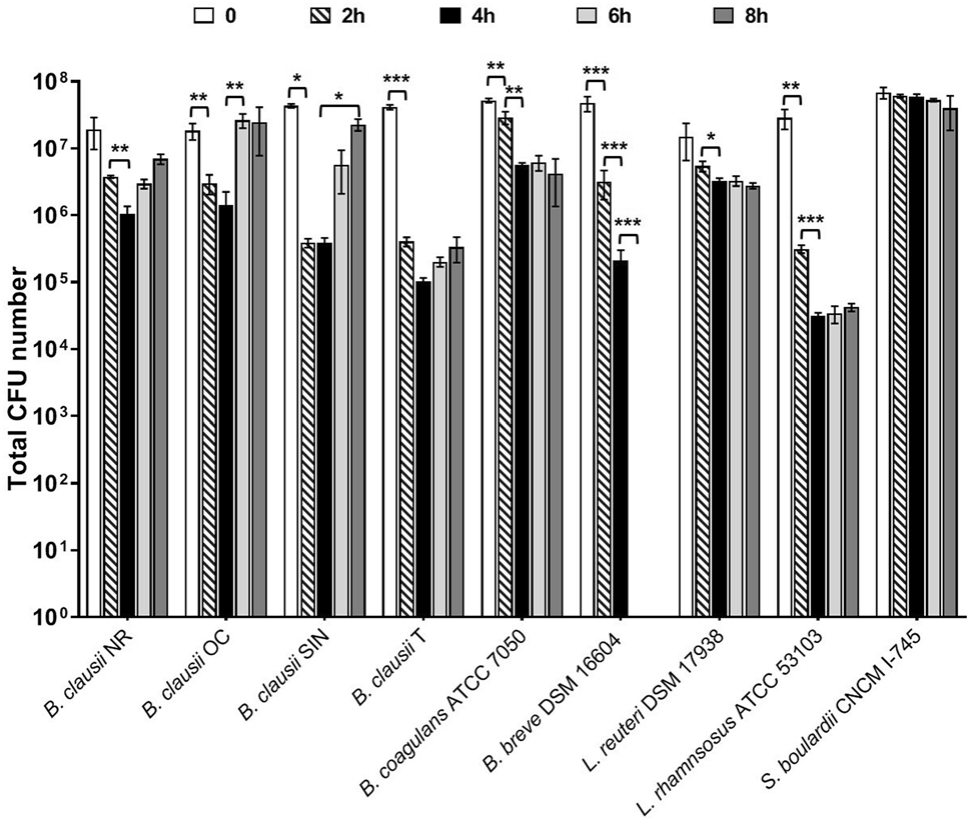
Survival of probiotic strains in simulated intestinal juice. Microbial counts (total CFU number) were performed at the inoculum (0, open bars) and after 2 (diagonally hatched bars), 4 (closed bars), 6 (light grey bars), and 8 (dark grey bars) hours of incubation at 37 °C. Three independent biological replicates with two technical replicates each were performed. Data are expressed as the mean ± standard deviation. For each strain, ANOVA for repeated measures followed by Tukey HSD test was applied to compare the total CFU numbers obtained at each time point. *P < 0.05, **P < 0.01, ***P < 0.001.

The analyzed microbial strains exhibited a very different behavior in simulated intestinal fluid (Fig. 1). The total colony forming units (CFU) number of *B. clausii* NR and T started to decrease at 4 h (P < 0.01 compared to 2 h) and 2 h (P < 0.001 compared to time 0), respectively. For both strains, the number of CFU recorded after these time points was stable. After 8 h of incubation, a reduction in the total amount of cells compared to the inoculum was registered for both strains. The total CFU number of *B. clausii* OC decreased after 2 h of incubation in the juice (P < 0.01 compared to 0 min), while started to increase at 6 h (P < 0.01 compared to 4 h). Following an initial decrease at 2 h (P < 0.05 compared to time 0), *B. clausii* SIN was able to multiply in the fluid starting from 8 h of incubation (P < 0.05 compared to 4 h; Fig. 1) and only a slight reduction of 0.240-Log was observed at this time compared to the inoculum.

A decrease in the total number of *B. coagulans* and *L. reuteri* was registered at different times post inoculation (P < 0.01 for *B. coagulans* at 2 h and 4 h compared to 0 and 2 h, respectively; P < 0.05 for *L. reuteri* at 4 h compared to 2 h; Fig. 1). Interestingly, the total CFU number of *B. breve* progressively decreased in the simulated intestinal fluid (decrease of about 1 − Log, P < 0.001 at 2 h compared to 0 min and at 4 h compared to 2 h) and no residual living cells were obtained starting from 4 h of incubation (P < 0.001 at 6 h compared to 4 h). The total number of *L. rhamnosus* decreased after 2 h and 4 h (P < 0.01 at 2 h compared to 0 min and P < 0.001 at 4 h compared to 2 h). Afterwards, the amount of *L. rhamnosus* cells was stable over time with a reduction in the total amount of cells of 2.816 − Log at 8 h compared to the inoculum. Lastly, *S. boulardii* cells persisted in the juice for up to 8 h.

Overall, these results indicate that vegetative cells can behave differently in simulated intestinal fluid, depending on the tested microbial strain. Additionally, our findings highlight the ability of *B. clausii* NR and T, *B. coagulans*, *L. reuteri*, *L. rhamnosus*, and *S. cerevisiae* strains to survive in simulated intestinal conditions for up to 480 min. Interestingly, only *B. clausii* OC and SIN demonstrated the peculiar ability to grow even in the absence of external nutrient sources following an initial decrease in their number.

### In vitro adhesion of probiotic microbes to mucins

Adhesion of probiotic microorganisms to the gastrointestinal mucus is required for the interaction with host cells, thus allowing probiotics to exert their beneficial activities^6^. Therefore, we investigated the ability of *B. clausii*, *B. coagulans*, *B. breve*, *L. reuteri*, *L. rhamnosus*, and *S. boulardii* strains to adhere to mucins by using a microplate assay^16^.

**Figure 2:**
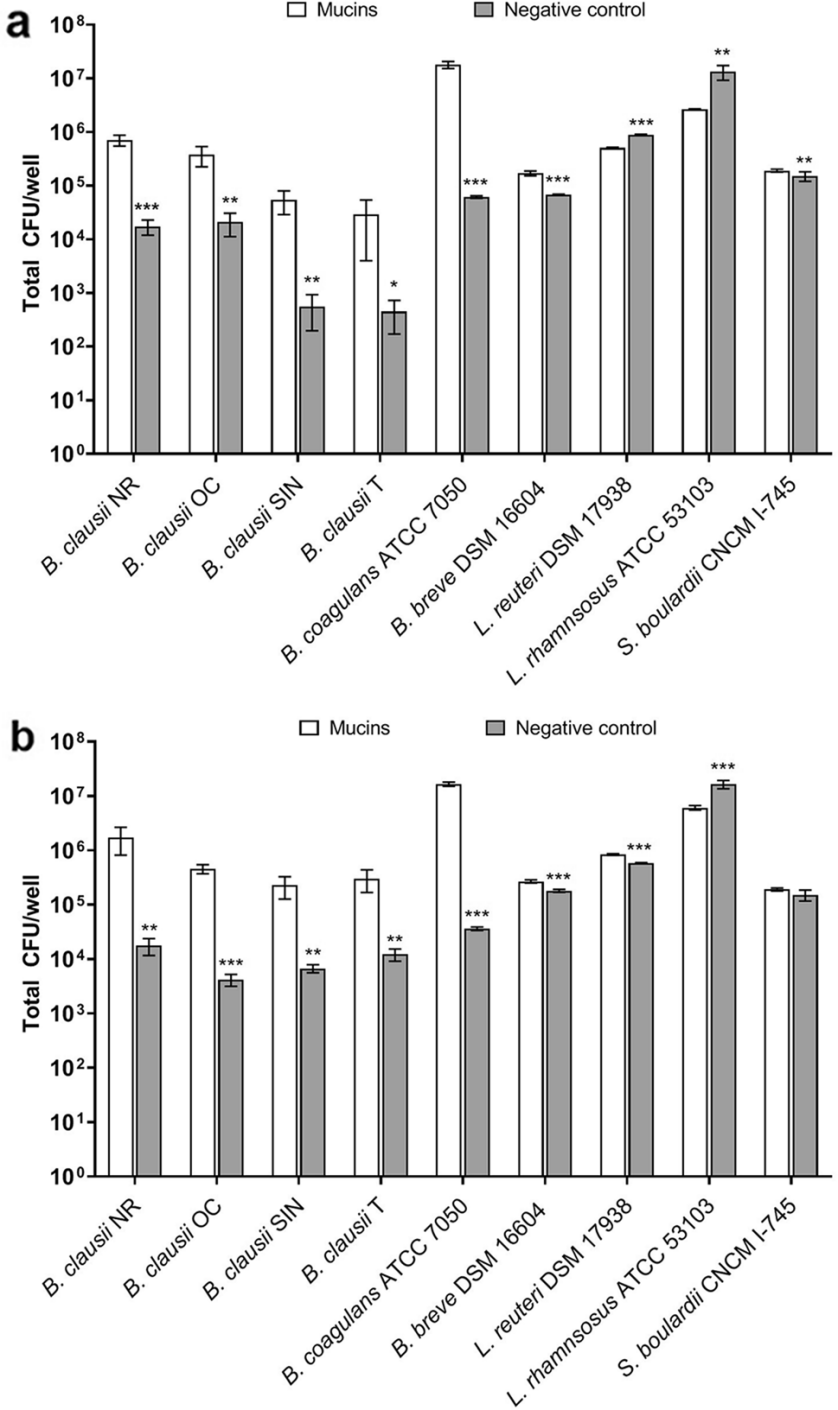
Adhesion of probiotic microbes to porcine mucins. (**a**) Amount of cells (CFU/well) mechanically extracted from agar containing mucins (open bars) and from agar without mucins (i.e. negative control, grey bars) after incubation at 37 °C in aerobiosis. (**b**) Amount of cells (CFU/well) mechanically extracted from agar containing porcine mucins (open bars) and from agar without mucins (i.e. negative control, grey bars) after incubation at 37 °C in anaerobiosis. Three independent biological replicates with two technical replicates each were performed. Data are expressed as the mean ± standard deviation. For each strain, the two-tailed Student’s t-test was used to compare the CFU/well obtained on mucins and negative control wells. *P < 0.05, **P < 0.01, ***P < 0.001.

For all *B. clausii* strains, the CFU/well obtained after incubation on mucins in aerobic conditions was significantly higher than that of the negative controls (P < 0.001 for strain NR; P < 0.01 for *B. clausii* OC and SIN; P < 0.05 for *B. clausii* T; Fig. 2a). As shown in Fig. 2b, the *B. clausii* strains were also able to adhere to mucins following incubation in anaerobic atmosphere (P < 0.01 for *B. clausii* NR, SIN, and T; P < 0.001 for *B. clausii* OC compared to negative control). Different behaviors were observed with the other microbial strains. *B. coagulans* and *B. breve* were able to adhere to mucins in both aerobic (P < 0.001; Fig. 2a) and anaerobic atmosphere (P < 0.001; Fig. 2b). On the other hand, *L. rhamnosus* was unable to bind mucins in both conditions, since the total CFU/well recovered from the negative controls was greater than that obtained from mucins (P < 0.01 in aerobic atmosphere and P < 0.001 in anaerobic atmosphere; Fig. 2a,b). While *L. reuteri* did not adhere to mucins in aerobiosis (Fig. 2a), its adhesion to mucins was substantially increased in anaerobic atmosphere (P < 0.001; Fig. 2b). Conversely, *S. boulardii* was found to adhere to mucins in aerobic atmosphere (P < 0.01; Fig. 2a) but not in anaerobic conditions (Fig. 2b).

### Production of β-galactosidase by probiotics

Administration of β-galactosidase-producing probiotics can be advantageous in people affected by lactose intolerance for reducing symptoms caused by lactose malabsorption^10^. Therefore, we wondered whether *B. clausii*, *B. coagulans*, *B. breve*, *L. reuteri*, *L. rhamnosus*, and *S. boulardii* could produce β-galactosidase.

**Figure 3:**
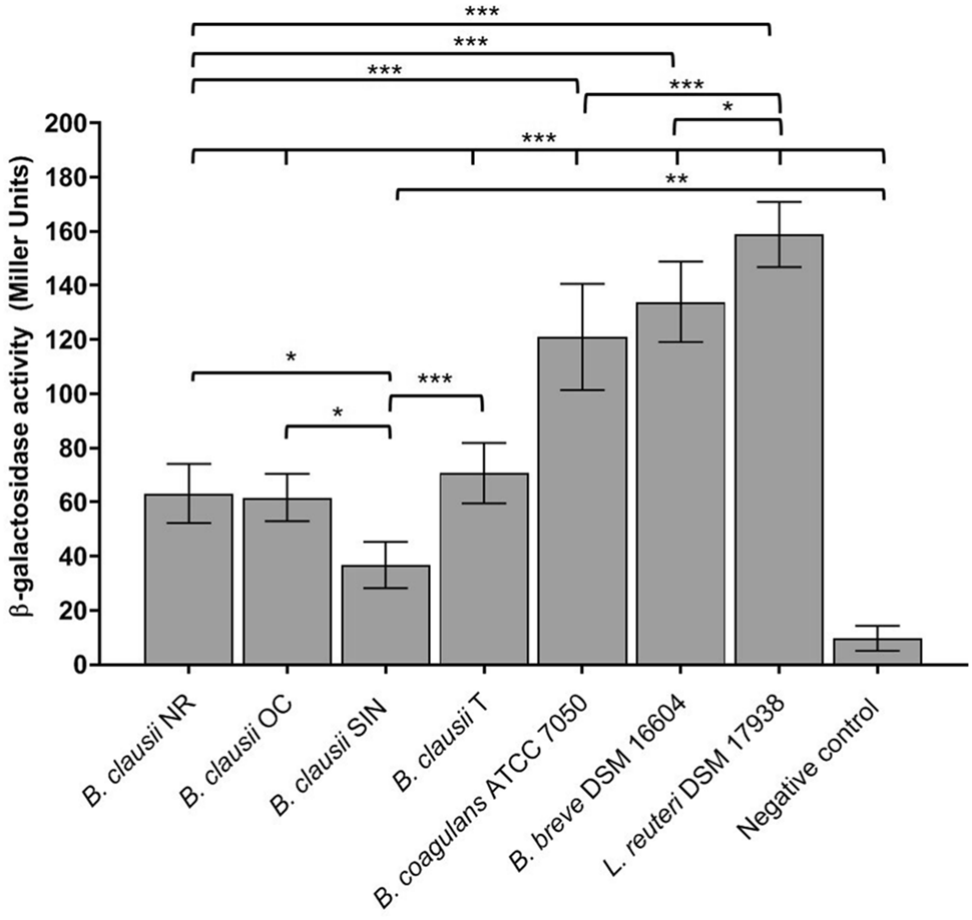
Quantification of the β-galactosidase activity (Miller Units) produced by probiotic microbes that resulted able to synthesize the enzyme on solid media. *P. mirabilis* ATCC 12453 was used as negative control in the assay (i.e. negative control). Three independent biological replicates with two technical replicates each were performed. Data are expressed as the mean ± standard deviation. The ANOVA for independent data followed by Tukey HSD test was applied. *P < 0.05, ***P < 0.001.

All *B. clausii* strains, *B. coagulans*, *B. breve*, and *L. reuteri* strains were able to grow on M63 minimal medium and to degrade X-Gal on M63 and LB agar plates, producing blue colonies (data not shown). In contrast, *L. rhamnosus* and *S. boulardii* strains were unable to grow on the minimal medium and formed white colonies on LB agar plates, thus resulting negative for β-galactosidase. The enzymatic activity produced by positive strains was quantified by using the Miller’s method^17^. As shown in Fig. 3, the amount of β-galactosidase produced by *B. clausii*, *B. coagulans*, *B. breve*, and *L. reuteri* strains was significantly higher than that of the negative control (P < 0.01 for *B. clausii* SIN; P < 0.001 for *B. clausii* NR, OC, and T, *B. coagulans*, *B. breve*, and *L. reuteri*), thus confirming the ability of these strains to synthetize β-galactosidase. Interestingly, *L. reuteri* produced the highest amount of enzyme compared to the other β-galactosidase producing strains (P < 0.05 compared to *B. breve*; P < 0.001 compared to the other strains; Fig. 3). *B. coagulans* and *B. breve* synthetized higher enzyme levels than the *B. clausii* strains (P < 0.001). The enzymatic activity detected for *B. clausii* SIN was significantly lower compared to the other *B. clausii* strains (P < 0.05 compared to *B. clausii* NR and OC; P < 0.001 compared to *B. clausii* T).

Overall, these findings highlight the production of β-galactosidase by the analyzed probiotic *B. clausii*, *B. coagulans*, *B. breve*, and *L. reuteri* strains.

### Production of catalase and superoxide dismutase by probiotic microbes

The production of catalase (CAT) and superoxide dismutase (SOD) by probiotic microorganisms is thought to help the host in reducing oxidative stress^8^. The ability of *B. clausii*, *B. coagulans*, *B. breve*, *L. reuteri*, *L. rhamnosus*, and *S. boulardii* to produce CAT and SOD was evaluated by quantifying these enzymatic activities in cell lysates and culture supernatants collected from actively replicating cells grown in BHIG.

**Figure 4:**
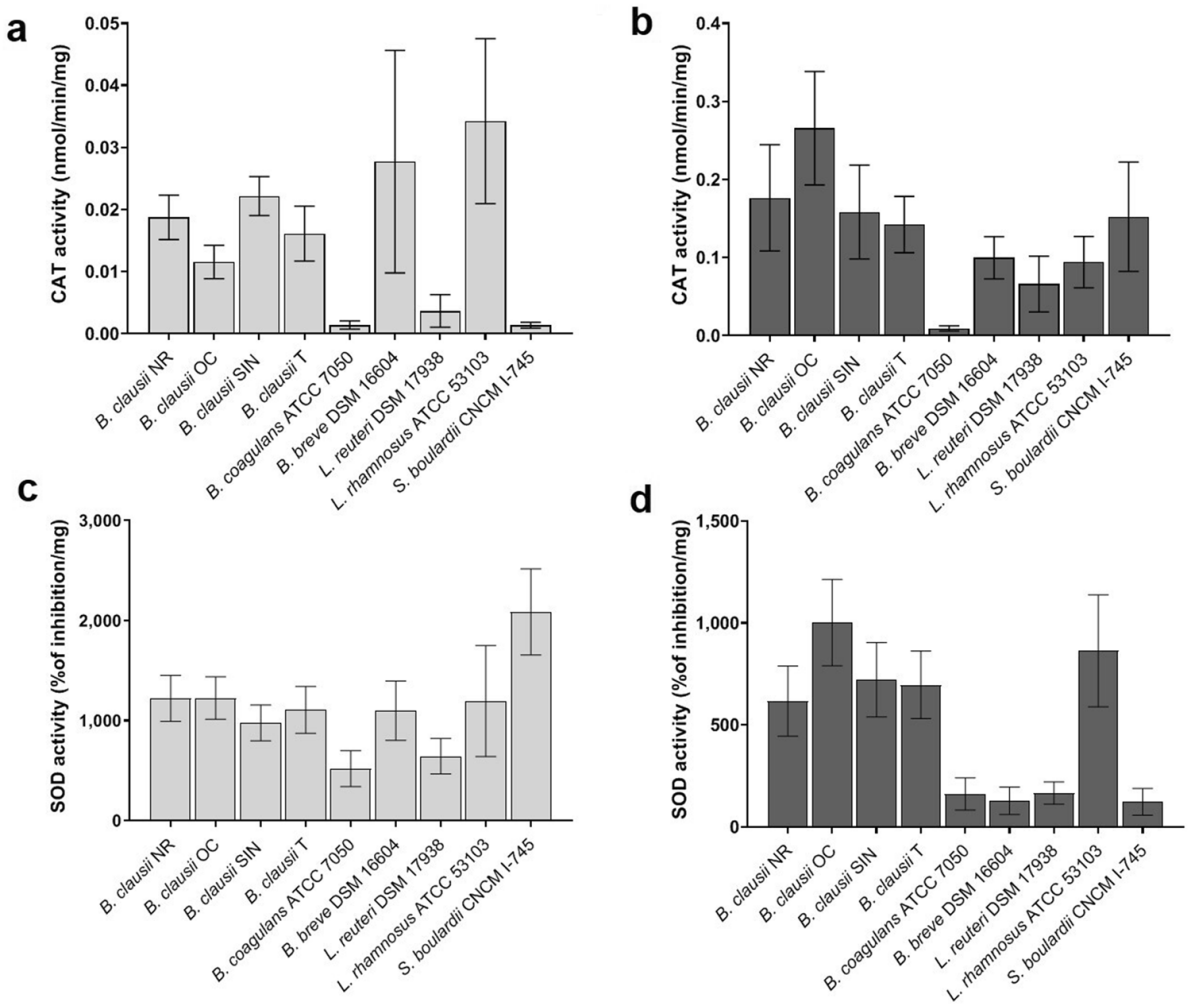
Quantification of catalase (CAT) and superoxide dismutase (SOD) activities produced by probiotic strains. (**a**) CAT activity (nmol/min/mg) determined in cell lysates (light grey bars). (**b**) CAT activity (nmol/min/mg) determined in culture supernatants (dark grey bars). (**c**) SOD activity (% of inhibition/mg) determined in cell lysates (light grey bars). (**d**) SOD activity (% of inhibition/mg) determined in culture supernatants (dark grey bars). Three independent biological replicates with two technical replicates each were performed. Data are expressed as the mean ± standard deviation. The ANOVA for independent data followed by Tukey HSD test was applied.

For all the strains, CAT and SOD activities were detected in both cellular compartments. The CAT levels were found to be higher in culture supernatants than in cell lysates (Fig. 4a,b).

When CAT activity was quantified in cell lysates (Fig. 4a), *L. rhamnosus* showed the highest levels of intracellular CAT (P < 0.05 compared to *B. clausii* NR, P < 0.01 compared to *B. clausii* T, P < 0.001 compared to *B. clausii* OC, *B. coagulans*, *L. reuteri*, and *S. boulardii*).

In culture supernatants (Fig. 4b), the highest CAT levels were detected for *B. clausii* OC (P < 0.05 compared to *B. clausii* SIN, P < 0.01 compared to *S. boulardii* and *B. clausii* T, and P < 0.001 compared to *B. breve L. reuteri*, and *L. rhamnosus*).

As shown in Fig. 4c,d, the SOD activity was generally higher in cell lysates than in culture supernatants. When SOD was quantified in cell lysates (Fig. 4c), the highest activity was obtained for *S. boulardii* (P < 0.001 compared to the other strains). By quantifying the SOD activity in culture supernatants (Fig. 4d), the highest SOD activity was measured for *B. clausii* strains and *L. rhamnosus* (P < 0.001 compared to the other strains). Significant differences were found in the extracellular SOD levels among the *B. clausii* strains. In fact, *B. clausii* OC showed higher SOD activity compared to NR and T (P < 0.01 and P < 0.05, respectively).

Taken together, our results indicate that all the tested strains are sufficiently equipped of antioxidant enzymes that can help in reducing oxidative stress in the host.

### Production of riboflavin and D-lactate by probiotic microbes

Probiotic microorganisms able to secrete riboflavin are attractive resources that can be useful to compensate host deficiency for this vitamin. Only *B. clausii*, *B. coagulans*, and *L. rhamnosus* strains were found able to produce riboflavin (P < 0.001 compared to the negative control, Table 1). *B. coagulans* was found to produce the highest level of riboflavin compared to the other strains (P < 0.001). These results highlight the ability of *B. clausii* NR, OC, SIN, and T, as well as of *B. coagulans* and *L. rhamnosus* strains to actively secrete vitamin B2 during growth.

**Table 1:** Amount of riboflavin produced by probiotic strains.

Strains	Riboflavin (ng/ml)
*Bacillus clausii* NR	22.26 ± 2.71
*Bacillus clausii* OC	25.50 ± 1.98
*Bacillus clausii* SIN	25.99 ± 4.83
*Bacillus clausii* T	25.33 ± 5.44
*Bacillus coagulans* ATCC 7050	324.63 ± 78.20
*Lacticaseibacillus rhamnosus* ATCC 53103	99.80 ± 18.73

It has been suggested that administration of d-lactate producing strains should be carefully considered in patients at risk of developing d-lactic acidosis^12,14^.

**Figure 5:**
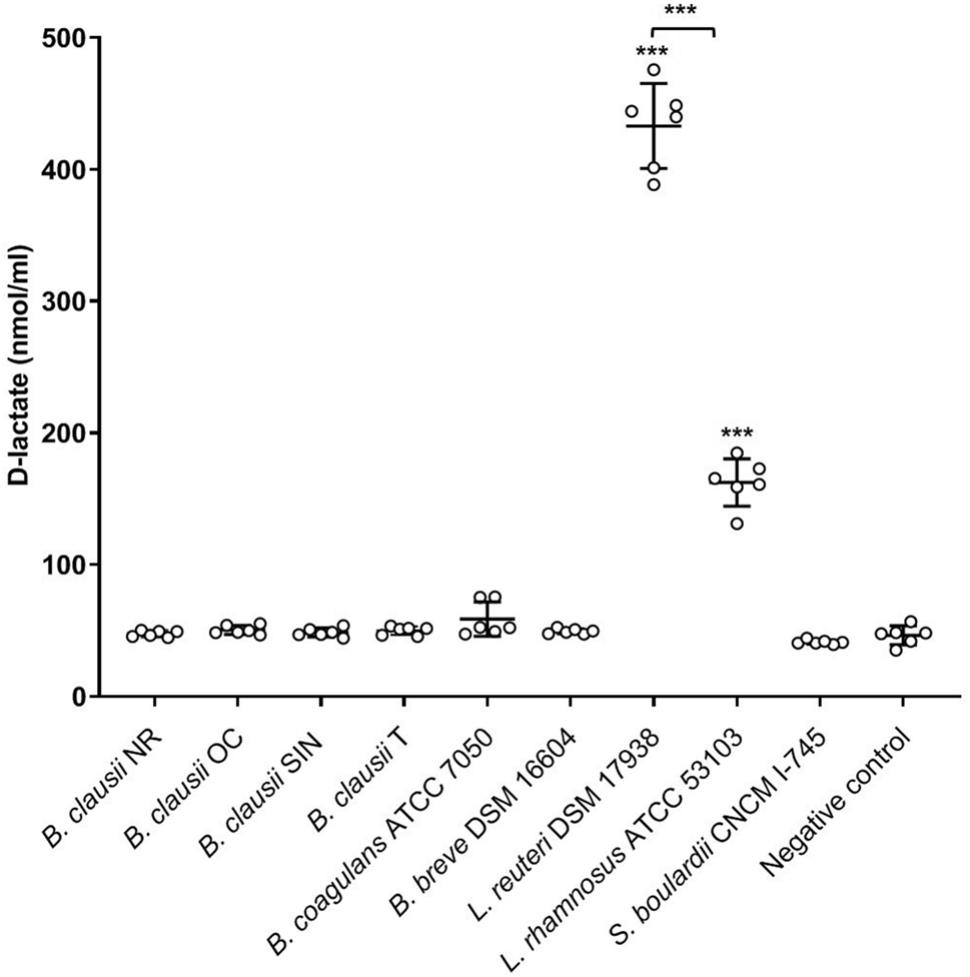
Production of d-lactate by probiotic microbes. Scatter plot of d-lactate concentration (nmol/ml) values determined in deproteinized culture supernatants of each microbe. Deproteinized BHIG was used as negative control in the assay (i.e. negative control). Three independent biological replicates with two technical replicates each were performed. The mean ± standard deviation was also shown. The ANOVA for independent data followed by Tukey HSD test was applied. ***P < 0.001.

As shown in Fig. 5, only *L. reuteri* and *L. rhamnosus* were found able to secrete d-lactate (P < 0.001 compared to the negative control), with *L. reuteri* producing significantly more d-lactate than *L. rhamnosus* (P < 0.001).

## Discussion

The ability to survive in the presence of bile and adhere to the intestinal mucus are considered key properties that orally administered probiotics should widely possess^6,7^. Alkaline artificial fluids containing bile are commonly adopted as models for evaluating the potential behavior of probiotic microorganisms in the intestinal environment^18–22^. Since the gastrointestinal tract is covered by mucus, mainly formed by mucins^6,23^, incubation of microbes on agar containing mucins in microplates represents a simple and reliable method for investigating their mucin-binding ability and has been adopted in several studies^15,24–26^.

In accordance with its documented bile salt-tolerance and ability to resist to alkaline pH^27^, *S. boulardii* CNCM I-745 well survived in the artificial intestinal fluid for up to 8 h. In the artificial intestinal fluid, our results for *B. breve* DSM 17938, *L. reuteri* DSM 17938, and *L. rhamnosus* ATCC 53103 are in line with evidences showing that *Bifidobacterium* and *Lactobacillus* species exhibit variable resistance to simulated intestinal conditions depending on the species and strain tested^19^. For this reason, bile-salt adapted strains and several microencapsulation techniques have been developed to increase the ability of these bacteria to tolerate harsh conditions and survive in the small intestine^28^.

The finding that *B. clausii* OC and SIN were able to replicate in the artificial intestinal juice without nutrients lead us to hypothesize that these strains had used the nutrients released by dead cells for multiplication. This behavior agrees with previous studies in which we showed that the spore mixture of strains NR, OC, SIN, and T can germinate and vegetative cells multiply in both the simulated intestinal fluid and human intestine^22,29^. It is important to underline that the tolerance of *B. clausii* and *B. coagulans* to simulated intestinal conditions can be improved by administering these strains as spores, which are generally contained in commercial products.

Since oxygen levels progressively decrease along the intestine^30^, we tested the ability of the selected microorganisms to interact with mucins in both aerobic and anaerobic atmosphere. The results obtained for *B. clausii* NR, OC, SIN, and T and *B. coagulans* ATCC 7050 could be expected, since genomic analysis of these *B. clausii* strains and of the *B. coagulans* S-lac strain revealed the presence of three mucus binding proteins and type-IV filamentous adhesins, respectively^31,32^. In addition, a role of flagellin, the main flagellar filament-component, in mucin adhesion was demonstrated for a probiotic *Bacillus cereus* strain^33^. In line with studies highlighting the adhesive properties of different *B. breve* strains^34,35^, *B. breve* DSM 16604 was found to adhere to mucins. Although the factors involved in *B. breve* mucin-adhesion are not fully clarified, type IVb tight adherence pili required for glycoprotein interaction have been shown to be essential for the in vivo murine gut colonization^34^.

Among *Lactobacillus* species, adhesive properties are extremely strain-specific and depend on the presence of specific adhesion factors^6,7,23,36–39^. The finding that *L. rhamnosus* ATCC 53103 was unable to bind mucins correlates with a previous study indicating that some *L. rhamnosus* strains isolated from different formulations were severely compromised in mucin-binding ability^40^. *L. reuteri* DSM 17938 and *S. boulardii* CNCM I-745 adhesion to mucins appeared to be influenced by oxygen levels, in line with their oxygen requirement.

Lactose intolerance is a clinical condition caused by β-galactosidase deficiency that results in the inability to degrade lactose contained in milk and derivatives^10,41^.

The ability to produce β-galactosidase is widely distributed among the *Bifidobacterium* and *Lactobacillus* genera and some strains were shown to bring clinical benefits in people affected by lactose intolerance and malabsorption^41–44^. However, the production of β-galactosidase by bacteria of these genera appeared strain-specific^43^. The production of this enzyme was also documented for some *B. coagulans* strains^45,46^.

In line with the evidence that many *L. reuteri* strains are able to synthesize considerable amounts of β-galactosidase^47,48^, the highest level of this enzyme was evidenced for *L. reuteri* ATCC DSM 17938. Herein we demonstrate that *B. clausii* can produce β-galactosidase. Among the tested *B. clausii* strains, we found that strain SIN produced lower levels of the enzyme. This result agrees with previous studies indicating that the analyzed *B. clausii* strains exhibit some quantitative differences in their proteome^49,50^. Overall, our results suggest that the tested *B. clausii*, *B. coagulans*, *B. breve*, and *L. reuteri* strains can potentially be useful in reducing the symptomatology associated with lactose malabsorption, thus providing beneficial effects in individuals affected by lactose intolerance.

Oxidative stress is caused by massive ROS accumulation and is associated with nucleic acid, protein, and lipid modifications, thus leading to cell apoptosis, cellular aging and to the development of several chronic diseases^9,51^. CAT and SOD play a crucial role as antioxidant enzymes, being involved in hydrogen-peroxide and superoxide radical scavenging, respectively^9^. Due to the production of antioxidant enzymes, a role of probiotics in the prevention of oxidative stress and several ROS-linked diseases has been proposed in the last decades^8,9,52^. The finding that the tested *Lactobacillus* strains showed catalase activity was unexpected, since lactobacilli are generally catalase negative. We can only hypothesize that the adopted assay detects a reduction in the amount of hydrogen peroxide due to the activity of non-catalase enzymes. Other studies will be required to address whether these strains possess an alternative enzymatic activity able to degrade hydrogen peroxide. The results obtained with *B. clausii*, *B. coagulans*, *B. breve*, *L. reuteri*, *L. rhamnosus*, and *S. boulardii* strains indicate that their administration could be helpful in reducing or alleviating ROS accumulation, thus preventing oxidative stress. In particular, the finding that the levels of secreted CAT were higher than those found in the cytoplasm suggests that the analyzed microbes could contribute to counteract extracellular hydrogen peroxide accumulation in vivo, thus exerting a cytoprotective effect.

The B-group and water-soluble vitamin riboflavin is the precursor of flavin adenine mononucleotide and flavin adenine dinucleotide, which act as coenzymes in several redox reactions and are involved in the metabolism of carbohydrates, proteins, lipids, other vitamins, and ketone bodies^53–55^. Ariboflavinosis due to dietary inadequacy or gut microbiota dysbiosis can be responsible for a variety of conditions, including cataract, childhood neuropathy, anemia, hypertension, certain types of cancer, and diabetes mellitus^53,54^. For this reason, the use of riboflavin-producing strains and/or administration of probiotic formulations supplemented with exogenous riboflavin can represent a promising source to prevent or ameliorate host ariboflavinosis.

The ability of *B. clausii* NR, OC, SIN, and T to produce riboflavin confirms a previous study in which these strains were tested for vitamin B2 production using an auxotrophic *B. cereus* mutant^56^. In accordance with the presence of the riboflavin-metabolic pathway in the *B. coagulans* genome^57,58^, we found that *B. coagulans* ATCC 7050 was a very good producer of riboflavin. The detection of riboflavin in *L. rhamnosus* ATCC 53103 supernatants agrees with previous data reported for this strain^59^.

d-lactic acidosis is a neurological disorder typical of patients affected by short bowel syndrome (SBS) and due to massive d-lactate accumulation in the bloodstream^15,60,61^. The administration of d-lactate producing probiotics in the development of d-lactic acidosis has been widely debated and currently appears to be a controversial issue^1,62–65^. However, some authors proposed that d-lactate producing strains should not be administered to patients at risk of developing d-lactic acidosis, including those with SBS and neonates^12,14^. In accordance with the presence of d-lactate dehydrogenase in species belonging to the *Lactobacillus* genus^15,59,66,67^, we only detected d-lactate in cell culture supernatants of *L. reuteri* and *L. rhamnosus* strains.

In conclusion, our findings demonstrate that the investigated probiotic strains are characterized by distinct biological properties in vitro. On the other hand, the only use of in vitro analyses cannot be conclusive to explain the behavior probiotic strains exhibit in vivo, due to the complexity of the gastrointestinal tract. However, we believe that studies investigating the in vitro properties of microbes isolated from commercial products can be of translational relevance, since supporting clinical research in the comprehension of the beneficial effects these formulations may provide in vivo.

## Methods

### Bacterial strains and culture conditions

Strains used in this study are listed in Table 2. Microbes were isolated from commercial formulations purchased at the local pharmacy. The isolated species were identified by Matrix-Assisted Laser Desorption Ionization–Time of Flight Mass Spectrometry (MALDI-TOF MS) and the strain names as declared by manufactures on the product labels adopted throughout the study. The multi-strain formulation Enterogermina (100 μl) was seeded on Brain Heart Infusion (BHI; Thermo Fisher Scientific, USA) agar plates supplemented with 50 μg/ml rifampicin, 50 μg/ml chloramphenicol, 200 μg/ml streptomycin, or 25 μg/ml tetracycline for selective isolation of *B. clausii* NR, OC, SIN, and T, respectively. The *B. clausii* strains used in this study are characterized by a low level of intra-specific genome diversity but showed strain-specific proteomic signaturers^31,48,49^. For mono-strain formulations, microbial isolation was performed as follows. *B. coagulans* was isolated by seeding 100 μl of Lactò Più on Trypticase Soy agar containing 5% horse blood and plates were incubated at 37 °C for 48–72 h. Neovaxitiol (100 μl) was streaked on Bifidus selective agar (Merck KGaA, Germany) containing 0.116 g/l of BSM Supplement (Merck KGaA) and grown for 48–72 h in anaerobic atmosphere by using Thermo Scientific™ AnaeroGen™ Compact (Thermo Fisher Scientific) for isolating *B. breve*. For *L. reuteri* and *L. rhamnosus* isolation, 5 drops of Reuflor and Dicoflor were streaked on De Man, Rogosa, Sharpe agar (Thermo Fisher Scientific) and plates were incubated at 37 °C for 48 h in 5% CO_2_ by using Thermo Scientific™ CO2Gen™ Compact (Thermo Fisher Scientific). For *S. cerevisiae* isolation, one capsule of CODEX was dissolved in 5 ml of sterile phosphate buffered saline (PBS, 1 M KH_2_PO_4_, 1 M K_2_HPO_4_, 5 M NaCl, pH 7.2) and 100 μl were seeded on SABOURAUD-2% dextrose agar (Merck KGaA). Plates were incubated at 30 °C for 48–72 h. Microorganisms were maintained as stocks at − 80 °C until use. When required, liquid cultures were performed in BHI supplemented with 1% (w/v) glucose (BHIG) or Luria Bertani (LB, Carlo Erba, Italy) at 37 °C.

**Table 2:** Probiotic strains used in this study and commercial formulations from which they were isolated.

Strain declared on the product label	Product	Manufacturer
*Bacillus clausii* NR	Enterogermina	Sanofi, France
*Bacillus clausii* OC	Enterogermina	Sanofi, France
*Bacillus clausii* SIN	Enterogermina	Sanofi, France
*Bacillus clausii* T	Enterogermina	Sanofi, France
*Bacillus coagulans* ATCC 7050	Lactò Più	RECORDATI S.p.A., Italy
*Bifidobacterium breve* DSM 16604	Neovaxitiol	IBSA Farmaceutici, Italy
*Limosilactobacillus reuteri* DSM 17938*	Reuflor	Italchimici S.p.A, Italy
*Lacticaseibacillus rhamnosus* ATCC 53103*	Dicoflor	AG Pharma S.r.l, Italy
*Saccharomyces boulardii* CNCM I-745	CODEX	Zambon Italia, Italy

*The nomenclature of these strains, originally reported as *Lactobacillus reuteri* DSM 17938 and *Lactobacillus rhamnosus* ATCC 53103 on the product labels, was updated according to the current nomenclature^69^.

### MALDI-TOF MS

For each microbe, well-isolated colonies were spotted on MALDI plates and overlaid with 1 μl of 70% ethanol, 1 μl of formic acid, and 1 μl of acetonitrile. After the addition of 1 μl of a-cyano-4-hydroxycinnamic acid matrix to each spot, plates were air-dried. The identification was performed by using the MALDI-TOF Microflex LT Mass Spectrometer (Bruker, USA). Spectra were acquired at a laser frequency of 60 Hz with an acquisition range from 1.960 to 20.000 Da, imported into the integrated MALDI Biotyper software (version 3.1, Bruker), and compared with reference spectra collected in the database. A score ≥ 2.00 indicated identification at the species level.

### Microbial behavior in simulated intestinal fluid

To prepare simulated intestinal fluid, 0.3% Oxgall bile salts (Merck KGaA) and 0.1% pancreatin (Merck KGaA) were dissolved in sterile 0.85% NaCl solution and adjusted to pH 8.0^20,21^. For each microbe, 100 µl of overnight cultures were inoculated in 5 ml of fresh BHIG and grown at 37 °C to OD_600_ of ~ 1.5. Cultures were centrifuged at 4500 rpm for 15 min at 4 °C and washed twice with sterile PBS. Supernatants were removed and microbial pellets suspended in 5 ml of simulated intestinal juice. Cultures were incubated at 37 °C for 0, 2 h, 4 h, 6 h, and 8 h in microaerophilic atmosphere by using a candle jar. At each time point, aliquots (100 μl) of the suspensions were serially diluted and seeded on solid media. The number of CFU was determined and the total CFU number contained in the juice calculated.

### Mucin adhesion in aerobic and anaerobic conditions

Microbial adhesion to mucins was assessed as described by Tsilia et al.^16^ with some modifications. Briefly, 100 µl of overnight cultures were inoculated in 25 ml of fresh BHIG and grown to an OD_600_ of ~ 1.5. Cultures were centrifuged at 4500 rpm for 10 min at 4 °C and pellets washed two times with sterile PBS. 500 µl of the suspensions were added to 48 well plates (Merck KGaA) containing 600 µl of mucin agar (pH 6.8), constituted by 5% (w/v) mucins from porcine stomach type II (Merck KGaA) and 1% (w/v) bacteriological agar. As negative controls, 500 µl of the same suspensions were inoculated on wells containing 600 µl of 1% (w/v) bacteriological agar. Plates were incubated at 37 °C for 90 min at 50 rpm in aerobic and anaerobic conditions. After incubation, the liquid phase was discarded and wells washed two times with 500 µl of PBS to remove loosely adhered cells. Microbial extraction was performed by mechanical method. The whole solid layers were aseptically transferred in 5 ml of physiological peptone solution and homogenized for 5 min. Aliquots were seeded on agar plates for quantification of adhered bacteria by the plate count method.

### Preparation of cell lysates and culture supernatants

Protein samples were prepared by inoculating microbial cells in 25 ml of BHIG. Cultures were grown at 37 °C to OD_600_ of ~ 1.8. Supernatants were separated from pellets by centrifugation at 10,000×*g*, collected, and filtered through 0.22 μm filters to completely remove cells. Microbial pellets were washed two times with sterile cold PBS and suspended in 1 ml of PBS. Cell suspensions were added with an equal amount of zirconia beads (0.1 mm of diameter) and lysed with a Bead Beater homogenizer by alternating 4 cycles of 1.0 min of lyses and 10 min of refrigeration in ice bath. Cell debris were removed by centrifugation at 10,000 rpm for 15 min at 4 °C and the soluble fractions collected. The protein content of cell lysates and culture supernatants was determined by using the BCA Protein Assay Kit (Thermo Fisher Scientific) according to the manufacturer’s instructions. Protein samples were stored at − 80 °C until use.

### Quantification of enzymatic activities

The activity of CAT and SOD were quantified in cell lysates and culture supernatants. CAT and SOD activities were measured by using the Catalase Assay kit (abcam, UK) and the Superoxide Dismutase Activity Assay kit (Colorimetric, abcam), respectively, following the manufacturer’s instructions.

Qualitative evaluation of β-galactosidase production was performed by inoculating microbial cells in 5 ml of LB broth containing 0.5 mM IPTG (Thermo Fisher Scientific) for 16 h at 37 °C. Microbial cultures (10 μl) were dropped on M63 synthetic medium (2 g/l (NH_4_)_2_SO_4_, 13.6 g/l KH_2_PO_4_, 0.5 mg/l FeSO_4_ × 7H_2_O, 1 mM MgSO_4_, 0.001 g/l thiamin, 10 g/l lactose, pH 7.0) and LB agar plates, both containing 40 µg/ml X-Gal (Merck KGaA) and 0.5 mM IPTG. Plates were incubated at 37 °C for 48–72 h. Quantification of the β-galactosidase activity was performed as described by Miller^17^. Briefly, microbial cells were inoculated in 5 ml of LB broth supplemented with 0.5 mM IPTG and grown at 37 °C to OD_600_ of ~ 0.5. Cells were collected by centrifuging 1 ml of each culture at 8000×*g* at 4 °C for 5 min and suspended in 1 ml of cold Z buffer (60 mM Na_2_HPO_4_ × 7H_2_O, 40 mM NaH_2_PO_4_ × H_2_O, 10 mM KCl, 1 mM MgSO_4_ × 7H_2_O, and 50 mM β-mercaptoethanol, pH 7.0). Cells permeabilization was performed by adding 20 μl of 0.1% (w/v) sodium dodecyl sulfate (Merck KGaA) and 40 µl of chloroform and by vortexing the tubes for 10 s. Samples (100 µl) were diluted in 900 μl of Z buffer and supplemented with 200 µl of 4 mg/ml orto-nitrofenil-β-galactopyranoside (ONPG, Merck KGaA). The reaction was conducted at 28 °C by monitoring the yellow color development and was stopped by adding 250 µl of 1 M Na_2_CO_3_. β-galactosidase activity was calculated by the equation (Eq. (1)):

1 $$\text{Miller units} =1000\times \frac{{\text{OD}}_{420 }-1.75{\times \text{OD}}_{550}}{\text{T}\times \text{V}{\times \text{OD}}_{600}},$$

where OD_420_ is the optical density measured at 420 nm, OD_550_ the optical density measured at 550 nm, OD_600_ the optical density measured at 600 nm, T the reaction time (expressed in min), and V the volume of culture assayed (expressed in ml). *Proteus mirabilis* ATCC 12453 and *Escherichia coli* K12 were used as negative and positive controls in the assays, respectively.

### Quantification of riboflavin

The amount of riboflavin in culture supernatants was determined by using the Enzyme-linked immunosorbent assay kit for vitamin B2 (Cloud-clone Corp., USA) according to manufacturer’s instructions. Sterile BHIG was used as negative control of the assay.

### Evaluation of D-lactate production

Before performing d-lactate quantification, 1 ml of culture supernatants was deproteinized by precipitation with 10% (v/v) TCA 6N^68^. The presence of d-lactate in supernatants was evaluated by using the colorimetric d-lactate Assay Kit (abcam) according to manufacturer’s instructions. Deproteinized BHIG was used as negative control.

### Statistical analysis

For all the experiments, three independent biological replicates with two technical replicates each were performed. Data were expressed as the mean ± standard deviation (S.D). Both statistical analyses and graphs were realized on GraphPad Prism version 8.0.2 (GraphPad Software Inc., USA, https://www.graphpad.com/scientific-software/prism/). For assessing the viability of each microbe in simulated intestinal juice, the one-way analysis of variance (ANOVA) for repeated measure followed by Tukey HSD test was applied to compare the total CFU numbers obtained at each time point. For mucin adhesion experiments, the two-tailed Student’s t-test was used to compare the CFU/well obtained for each strain on mucins and negative control wells. For the other assays, ANOVA for independent data followed by Tukey HSD test was used to separately compare the amount of molecules produced by each strain. A two-sided P-value (P) < 0.05 was considered significant.
